# Study of Involuntary Limb Movements as a Presenting Feature in Nonketotic Hyperglycemia

**DOI:** 10.7759/cureus.43579

**Published:** 2023-08-16

**Authors:** Srikant K Dhar, Asif D Jafri, Kaneez Fatima, Swati Samant, Sonam Samal, Sourav Maiti

**Affiliations:** 1 Department of Medicine, Institute of Medical Sciences (IMS) and SUM Hospital, Bhubaneshwar, IND; 2 Department of Medicine, SUM Ultimate Hospital, Bhubaneshwar, IND; 3 Department of Radiodiagnosis, All India Institute of Medical Sciences, Bhubaneswar, Bhubaneswar, IND; 4 Department of Ophthalmology, Institute of Medical Sciences (IMS) and SUM Hospital, Bhubaneswar, IND; 5 Department of Medicine, Institute of Medical Sciences (IMS) and SUM Hospital, Bhubaneswar, IND

**Keywords:** hyperintense basal ganglia, dyskinesia, hemichorea, hemiballismus, non-ketotic hyperglycemia

## Abstract

Background

Hyperglycaemia can rarely manifest as hemichorea/hemiballismus, which subsides with adequate control of blood sugar. Our study accounted for patients with abnormal, involuntary limb movements with high blood sugar, excluding other conditions leading to or mimicking such a clinical appearance. It is very important to identify such patients as chorea secondary to an underlying etiology like hyperglycemia, which can be cured.

Material & methods

This study was done in IMS & SUM Hospital for a duration of one year, from March 2019 to February 2020, with a total of 11 cases with abnormal limb movements with a blood sugar of 250 mg% and above.

Results

In this study, 36.36%( n=4) of patients were female, and 63.63% (n=7) were males. The mean age of the patients at presentation was 66.5 years. Eighteen point one percent (18.1%; n=2) of the patients showed hemiballismus, 36.3% (n=4) showed hemichorea, 18.1% (n=2) showed hemiathetosis, 9.1% (n=1) showed myoclonus, and 18.1% (n=2) showed hemiballismus with hemichorea. The mean duration to correct hyperglycemia was found to be 34 hours and the mean duration to correct abnormal limb movements was 90.54 hours. Eighty-one point eight percent (81.8%; n=9) of patients showed basal ganglia changes on brain imaging.

## Introduction

Non-ketotic hyperglycemia-induced hemichorea/hemiballismus is a rare metabolic syndrome that occurs secondary to hyperglycemia in a known diabetic and can also be the first presenting symptom of diabetes [1]. It is characterized by a triad of hemichorea-hemiballismus, hyperglycemia, and specific abnormalities that are restricted to the basal ganglia on neuroimaging [2,3]. Non-ketotic hyperglycemia is the most common metabolic cause of chorea/hemiballismus and it accounts for 1% of the total causes [4]. This has a very good prognosis with early diagnosis and treatment. There are several hypotheses regarding non-ketotic hyperglycemia causing hemichorea or hemiballismus. One such hypothesis is hyperviscosity of blood leading to striatal ischemia and disruption of the blood brain barrier (BBB). Another proposed mechanism is impaired cerebral autoregulation depleting gamma-aminobutyric acid (GABA) in basal ganglia. Despite the proposed hypotheses, the exact mechanism remains unclear. It is therefore interesting to know whether a threshold of blood sugar or glycated hemoglobin (HBA1c) level predisposes to such a condition. Our study accounted for patients with abnormal, non-specified, involuntary limb movements with high blood sugar, excluding those conditions possibly leading to or mimicking such a clinical appearance.

## Materials and methods

This study was undertaken at the Institute of Medical Sciences (IMS) and Sum Hospital for a duration of one year, from March 2019 to February 2020. A total of 11 cases with abnormal limb movements with blood sugar 250 mg/dl and above who presented to our outpatient department (OPD) or emergency department (ED) were analyzed retrospectively. Due to the retrospective nature of the study, the need for informed consent was waived. Cases with conditions that mimic such a presentation were excluded, e.g., patients with a history of seizure disorder, movement disorders, dyselectrolytemia, intracerebral space-occupying lesions, cerebral infarct/hemorrhage, sepsis, or toxin/drug overdose. Pregnant and pediatric age-group subjects were not included. They were admitted, and general and systemic clinical evaluation was thoroughly done. Random blood sugar (RBS), pH, blood routine, serum urea, creatinine, sodium, potassium, calcium, urine routine, HbA1C, and serum osmolarity were obtained. Brain imaging with computed tomography (CT) and/or magnetic resonance imaging (MRI) was done for each patient. All of them underwent an electroencephalogram (EEG) study. The management of all patients started with an intravenous titrated dose of regular insulin, which was later shifted to a maintenance dose of subcutaneous regular insulin. Haloperidol and clonazepam were given to six patients, and three patients required only haloperidol. Two patients responded to insulin alone. Time taken to achieve normoglycemia and to revert the abnormal movements were noted and assessed. The patients were followed up at the OPD with good compliance to treatment without recurrence and among them, four patients underwent follow-up neuroimaging at three months.

## Results

The baseline characteristics of patients at presentation have been shown in Table 1. Eleven patients were studied over a period of one year meeting the required criteria. Out of them, four were females and seven were males. Two patients were established cases of T2DM with poor glycemic control. Two cases were hypertensive on regular medications with adequate control. Two patients showed the presence of hypertension as well as diabetes. One patient had mild renal derangement, two patients had obstructive pulmonary disease (chronic obstructive pulmonary disease and asthma), and the remaining two patients directly presented with hyperglycemia and abnormal movements without any prior history of diabetes/other comorbidity. Four patients showed the involvement of both upper and lower limbs, whereas seven patients showed the involvement of the upper limbs alone. None of the patients showed isolated involvement of the lower limbs. Two had hemiballismus, four had hemichorea, two had hemi-athetosis, one had myoclonus, and two showed a combination of hemiballismus and hemichorea.

**Table 1:** Baseline characteristics of patients at presentation M- Males; F- Females; DM- Diabetes Mellitus; HTN- Hypertension; COPD- Chronic Obstructive Pulmonary Disease UL- Upper Limb; LL- Lower Limb

Age	Sex	Associated Comorbidity	Involvement	Abnormal movement
51	M	DM + HTN	UL + LL	Myoclonus
62	M	COPD	UL + LL	Hemichorea
67	F	DM + HTN	UL + LL	Hemichorea
82	M	Asthma	UL + LL	Hemiballismus + Hemichorea
70	M	Mild renal impairment	UL	Hemiballismus
49	F	DM	UL	Hemichorea
86	M	HTN	UL	Hemiballismus
79	M	HTN	UL	Hemiballismus + Hemichorea
73	F	DM	UL	Hemiathetosis
55	M	Absent	UL	Hemichorea
58	F	Absent	UL	Hemiathetosis

Table 2 depicts the mean values of various parameters of the patient. The mean age at presentation was 66.5 years (range 49-86 years). RBS values ranged from 350 to >700 mg/dl. The mean RBS value was found to be 542.81 mg/dl. Serum osmolarity ranged from 288.65 to 331.17 mosm/L on adequate treatment with regular insulin. The mean serum osmolarity was found to be 307.85 mg/dl. The duration to reverse hyperglycemia varied disproportionately with RBS and ranged from 12 to 72 hours (iv followed by s/c insulin). The mean duration to achieve normoglycemia was 34 hours. Duration to revert the abnormal movements ranged from 36 hours to 144 hours with the mean being 90.54 hours. Patients asserted a feeling of ‘well-being’. HbA1C varied from 13.6 to 18.8 units. The mean HBA1C was found to be 15.91.

**Table 2:** Mean values of various parameters RBS- Random Blood Sugar

Various parameters	Mean Values
Age at presentation	63 years
RBS	542.81 mg/dl
Serum Osmolarity	307.85mg/dl
Duration to achieve normoglycemia	34 hours
Duration to reverse limb movements	90.54 hours
HBA1C	15.91

Table 3 shows various associated complications in the patients. On neurological evaluation, one patient was found to be suffering from sensorimotor neuropathy, and three showed motor neuropathy. On further evaluation, three patients had diabetic retinopathy on fundoscopic screening. None of them was found to be in sepsis or acidosis. On brain radiological imaging, nine patients showed changes on CT/MRI, of which seven patients had unilateral involvement of basal ganglia, and two patients had bilateral involvement. No evidence of mass effect or edema was noted. Two patients had a normal scan. Follow-up scans of four patients at three months showed resolution of basal ganglia abnormalities. All 11 cases had a normal EEG study. No obvious correlation of comorbidities could be demonstrated.

**Table 3:** Various associated complications of the patients EEG- Electroencephalogram

COMPLICATIONS OF UNCONTROLLED BLOOD SUGAR		NO. OF PATIENTS
NEPHROPATHY		1
NEUROPATHY	SENSORIMOTOR	1
	MOTOR	3
RETINOPATHY		3
RADIOLOGICAL CHANGES	Basal ganglia involvement	9
	NORMAL	2
SEPSIS		0
ACIDOSIS		0
EEG ABNORMALITIES		0

**Figure 1:**
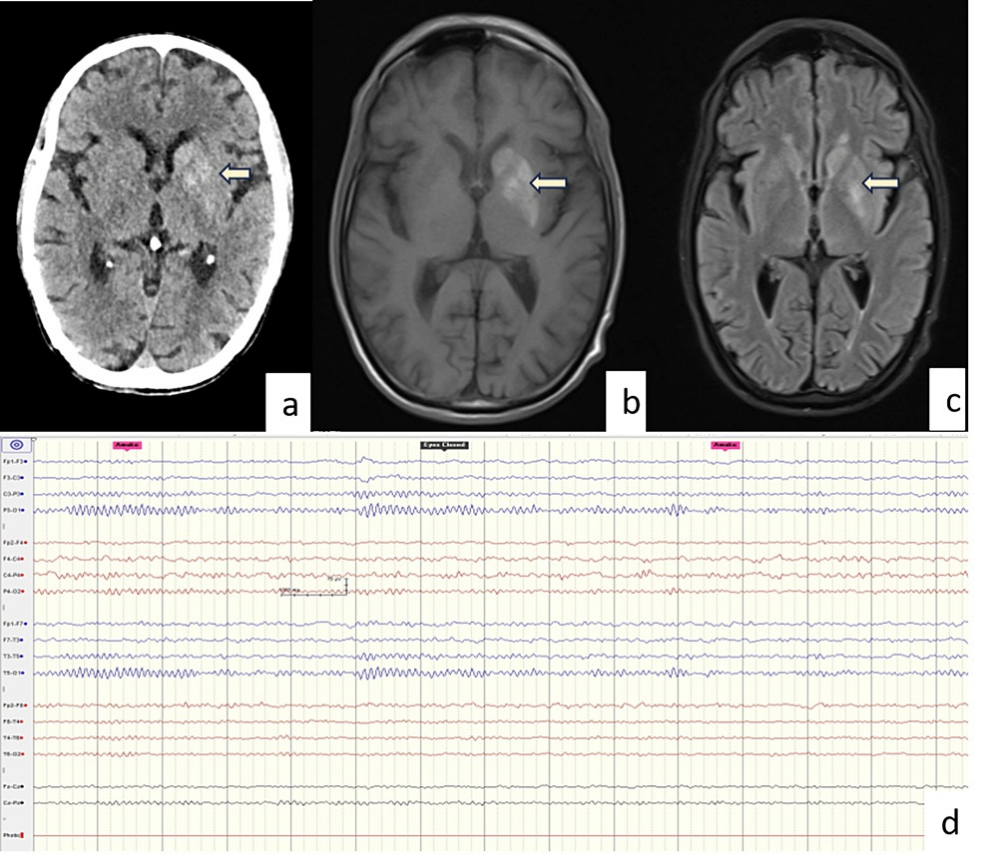
Neuroimaging (a) Brain CT of a 55-year-old male demonstrating hyperdensity (arrow) in the caudate and lentiform nucleus. MRI (axial) brain of the same patient showing hyperintensity (arrow) on T1W (b) as well as on T2W (c) imaging involving the caudate and lentiform nucleus. (d) Normal EEG of the same patient after the resolution of abnormal limb movement.

**Figure 2:**
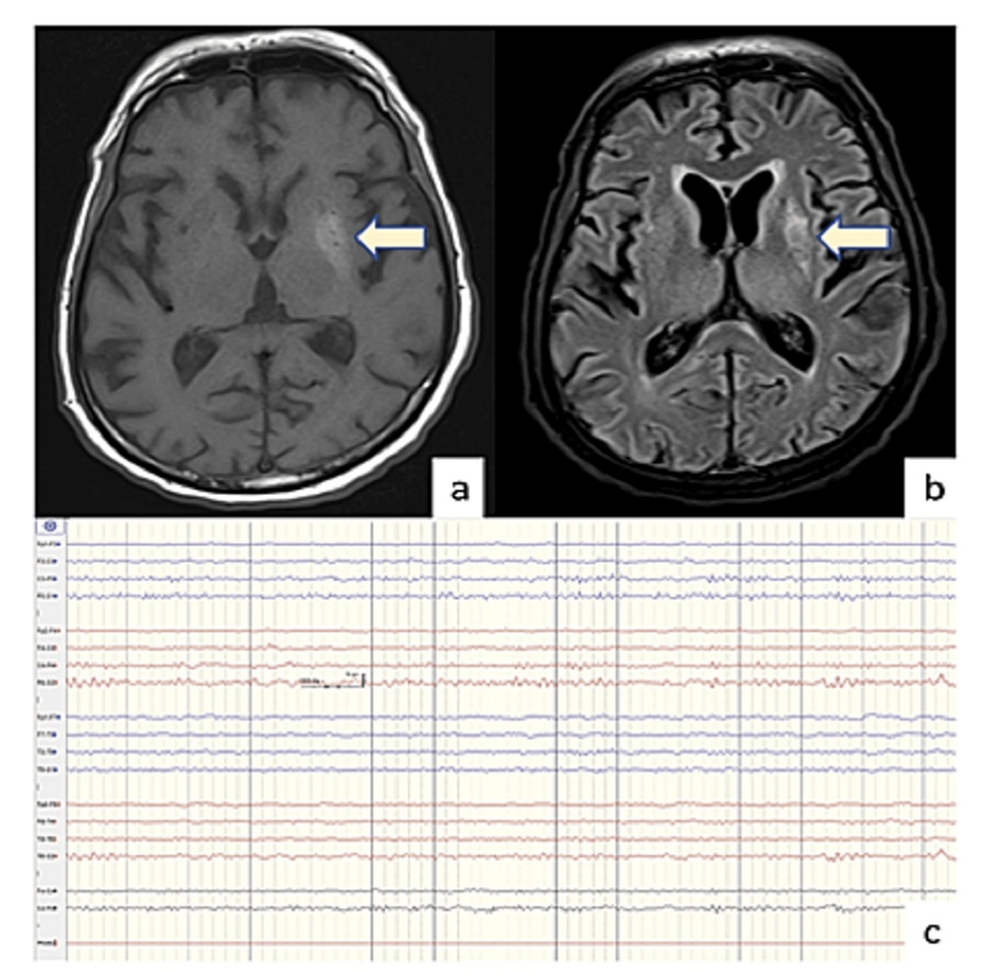
Neuroimaging (a) MRI (axial) brain of a 51-year-old female showing hyperintensity (arrow) on T1W (a) as well as onT2W (b) imaging (arrow) involving the left lentiform nucleus. (c) Normal EEG of the same patient after the resolution of abnormal limb movements.

**Figure 3:**
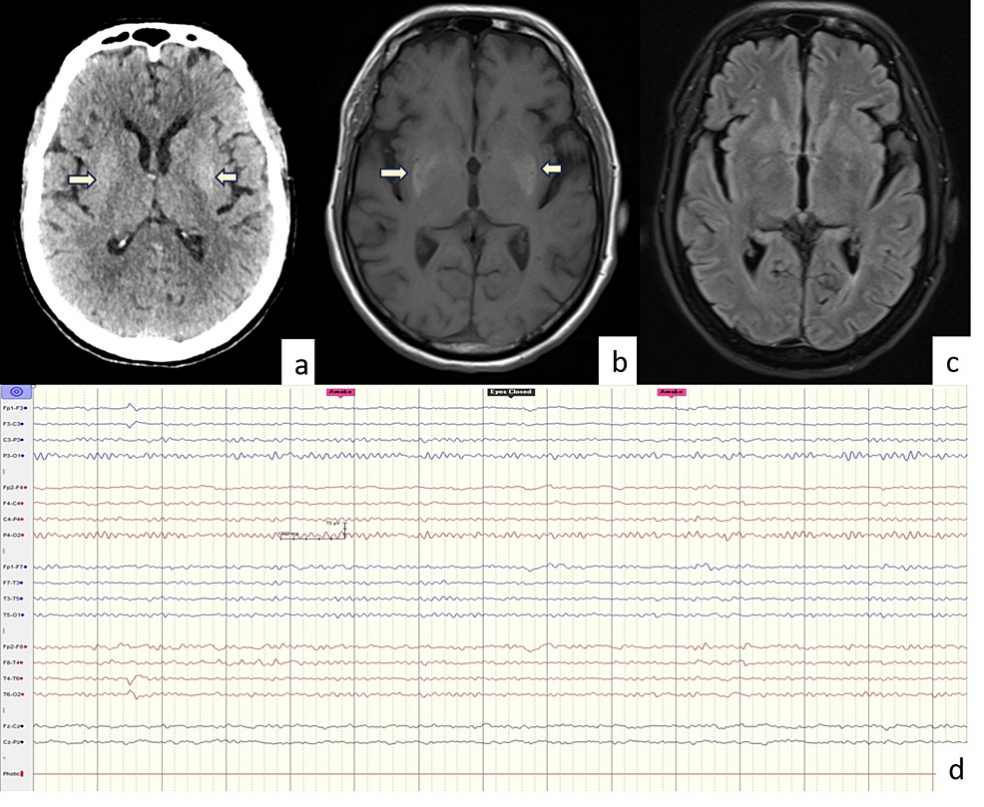
Neuroimaging (a) Brain CT of a 67-year-old male demonstrating hyperdensity (arrow) involving the bilateral lentiform nucleus. (b) MRI (axial) brain of the same patient showing symmetrical hyperintensity (arrow) in the bilateral lentiform nucleus on T1W (b) imaging. (d) Normal EEG of the same patient after the resolution of abnormal limb movements.

On brain radiologic imaging, nine patients showed changes on CT/MRI, of which seven patients had unilateral involvement of basal ganglia (Figures 1-2), and two patients had bilateral involvement (Figure 3). Two patients had a normal scan. All 11 cases had a normal EEG study.

## Discussion

The first report of hyperglycemia-induced hemiballismus was described by Bedwell in 1960 [5]. Since then, there have been several reports on hyperglycemia-induced chorea/hemiballismus. It is a rare complication of diabetes [6] and is also known as diabetic striatopathy or basal ganglia syndrome. This is an uncommon hyperglycemia-induced reversible basal ganglia abnormality manifesting radiologically as hyperdensity on CT scan and hyperdensity on MRI. The prevalence of diabetic striatopathy has been reported to be one in 100,000 [7]. In our study, nine patients out of 11 showed changes in the basal ganglia on neuroimaging. Due to its rare occurrence, it is often initially misinterpreted by physicians as an intracerebral bleed based on its appearance on brain imaging [4]. Oh et al. found that the male-to-female ratio was 1:1.76 by reviewing 53 cases reported in the Medline database from 1985 to 2001 [8]. This suggests that it occurs mainly in elderly women with uncontrolled diabetes. However, in our study, males were found to have a greater preponderance. This might be due to the fact that our study sample size was small. Neuroimaging showed T1W hyperintensity in the basal ganglia, with seven patients showing unilateral involvement and two patients showing bilateral involvement. No evidence of mass effect or edema was noted. Cases with similar changes on MRI imaging have already been reported in the literature [6-9].

There are also few reports of type 1 diabetes mellitus patients presenting with hemichorea/hemiballismus available [10-12]. However, in our study, none of the patients were found to be type 1 diabetic. There are also reports that showed an association between type 1 diabetes and Huntington’s chorea [12,13]. Chang X et al. reported a case where there was no change on neuroimaging [14]. Similarly, in our study, two out of 11 patients had not shown any changes.

The study done by Oh et al. and Chua et al. of patients presenting with abnormal limb movement and associated high blood sugar levels showed the following lab parameters - the mean serum glycemic level was 481.5 (range, 169-1,264) mg/dl, HbA1c level was 14.4% (range, 9.9-19.2%), and serum osmolarity was 305.9 (range, 291-335) mmol/kg [6,15]. Our lab parameters were very similar to those of the aforementioned studies.

Commonly occurring complications of diabetes like retinopathy was seen in three patients, neuropathy in four patients, and nephropathy in one patient. Sepsis was not seen in any of them.

In our study, insulin was administered along with haloperidol and clonazepam in six patients while three patients responded to haloperidol and insulin. Two patients did not require any anti-chorea medication. Thus, in some cases, restoration of euglycemia alone with insulin can lead to the resolution of symptoms. It was observed that it took around two to six days with insulin to show the reversal of any abnormal movements, and most people had a favorable outcome. Haloperidol has been associated with an increased risk of short-term mortality in elderly people [16]; however, we did not observe any such thing in our study.

Nakano N et al reported a drug-resistant case of hemichorea/hemiballismus in a diabetic patient treated by deep brain stimulation (DBS) [17]. However, in our study, all the patients were responsive to drugs and no patient needed DBS.

Limitations

The sample size of the study was less. Another limitation of this study was that follow-up imaging of the patients could not be done due to financial constraints.

## Conclusions

Non-ketotic hyperglycemia-induced abnormal movements are a benign condition mostly affecting the elderly. When a patient with uncontrolled blood sugar presents with any kind of abnormal unilateral limb movements, hyperglycemia should be among the differential to be considered. Immediate administration of insulin simultaneously clarifying other possible causes should be checked. With more exposure to such patients, the need for radiological imaging may be eliminated. However, electrolytes and fever profiles (if present) are indispensable. The mainstay of treatment is aggressive control of hyperglycemia with or without antipsychotics.

More studies with larger sample sizes are required to extract the varied cause of such presentation, the accuracy of treatment approach, and duration. Studies following known cases of diabetes may give strong support to the possibility of preventing this complication through good compliance with oral hypoglycemic agents. The use of 18-Fluoro-deoxyglucose positron emission tomography (FDG-PET)/single-photon emission computed tomography (SPECT) whenever possible may help know the status of cerebral glucose metabolism in hyperglycemia.

## References

[1] Mittal P (2011). Hemichorea hemiballism syndrome: the first presentation of type 2 diabetes mellitus as a rare cause of chorea. Iran J Radiol.

[2] Dong M, E JY, Zhang L, Teng W, Tian L (2021). Non-ketotic hyperglycemia chorea-ballismus and intracerebral hemorrhage: a case report and literature review. Front Neurosci.

[3] Wintermark M, Fischbein NJ, Mukherjee P, Yuh EL, Dillon WP (2004). Unilateral putaminal CT, MR, and diffusion abnormalities secondary to nonketotic hyperglycemia in the setting of acute neurologic symptoms mimicking stroke. AJNR Am J Neuroradiol.

[4] Ryan C, Ahlskog JE, Savica R (2018). Hyperglycemic chorea/ballism ascertained over 15 years at a referral medical center. Parkinsonism Relat Disord.

[5] Bedwell SF (1960). Some observations on hemiballismus. Neurology.

[6] Oh SH, Lee KY, Im JH, Lee MS (2002). Chorea associated with non-ketotic hyperglycemia and hyperintensity basal ganglia lesion on T1-weighted brain MRI study: a meta-analysis of 53 cases including four present cases. J Neurol Sci.

[7] Park G, Kesserwani HN (2022). A case report of diabetic striatopathy: an approach to diagnosis based on clinical and radiological findings. Cureus.

[8] Bekiesińska-Figatowska M, Romaniuk-Doroszewska A, Banaszek M, Kuczyńska-Zardzewiały A (2010). Lesions in basal ganglia in a patient with involuntary movements as a first sign of diabetes - case report and review of the literature. Pol J Radiol.

[9] Krishna S, Sodhi KS, Saxena AK, Singhi P, Khandelwal N (2015). Hyperdense basal ganglia in nonketotic hyperglycemia. J Emerg Med.

[10] Aquino JH, Spitz M, Pereira JS (2015). Hemichorea-hemiballismus as the first sign of type 1B diabetes during adolescence and its recurrence in the setting of infection. J Child Neurol.

[11] Lai PH, Tien RD, Chang MH (1996). Chorea-ballismus with nonketotic hyperglycemia in primary diabetes mellitus. AJNR Am J Neuroradiol.

[12] Podolsky S, Leopold NA, Sax DS (1972). Increased frequency of diabetes mellitus in patients with Huntington’s chorea. Lancet Lond Engl.

[13] Hashimoto K, Ito Y, Tanahashi H, Hayashi M, Yamakita N, Yasuda K (2012). Hyperglycemic chorea-ballism or acute exacerbation of Huntington's chorea? Huntington's disease unmasked by diabetic ketoacidosis in type 1 diabetes mellitus. J Clin Endocrinol Metab.

[14] Chang X, Hong W, Yu H, Yao Y (2017). Chorea associated with nonketotic hyperglycemia: a case report with atypical imaging changes. Medicine (Baltimore).

[15] Chua CB, Sun CK, Hsu CW, Tai YC, Liang CY, Tsai IT (2020). “Diabetic striatopathy”: clinical presentations, controversy, pathogenesis, treatments, and outcomes. Sci Rep.

[16] Schneeweiss S, Setoguchi S, Brookhart A, Dormuth C, Wang PS (2007). Risk of death associated with the use of conventional versus atypical antipsychotic drugs among elderly patients. CMAJ.

[17] Nakano N, Uchiyama T, Okuda T, Kitano M, Taneda M (2005). Successful long-term deep brain stimulation for hemichorea—hemiballism in a patient with diabetes. Case report. J Neurosurg.

